# Interactive effect of dietary calcium and phytase on broilers challenged with subclinical necrotic enteritis: 3. Serum calcium and phosphorus, and bone mineralization

**DOI:** 10.1016/j.psj.2020.04.012

**Published:** 2020-04-26

**Authors:** H.K. Zanu, S.K. Kheravii, N.K. Morgan, M.R. Bedford, R.A. Swick

**Affiliations:** ∗ School of Environmental and Rural Science, University of New England, Armidale, NSW 2351, Australia; † AB Vista, Marlborough, Wiltshire SN8 4AN, United Kingdom

**Keywords:** bone mineralization, high dietary calcium, necrotic enteritis, phytase, serum

## Abstract

Calcium is chelated by phytic acid and forms phytate-mineral complexes reducing Ca availability and the ability of phytase to hydrolyze phytate. An increased Ca concentration in the gut favors the activity of *Clostridium perfringens* (*C. perfringens*). Therefore, it was hypothesized that high dietary calcium with high dietary phytase would decrease serum Ca and P and bone mineralization during necrotic enteritis occurrence. A total of 768 one-day-old Ross 308 male chicks were randomly allocated to 8 treatments with 6 replicate pens, each housing 16 birds. A 2 × 2 × 2 factorial arrangement of treatments was applied: dietary Ca (0.6 or 1.0%), phytase (500 or 1,500 FTU/kg), and challenge (no or yes). Half of the birds (384) were challenged with *Eimeria* spp. on day 9 and *C. perfringens* strain EHE-NE18 on day 14 and 15. Blood was collected from 2 birds per pen to determine Ca, P, and parathyroid hormone in the serum. The middle toe, tibia, and femur were excised from 2 birds per pen on day 16 and 29 for determination of ash, breaking strength (**BS**), and mineral concentration. The challenge decreased (*P* < 0.05) serum Ca^+^ in birds regardless of dietary Ca level (day 16). There was a challenge × Ca interaction (*P* < 0.05) for tibial BS (day 16), with challenge being more severe in birds fed high Ca than low Ca diets. A challenge × phytase interaction (*P* < 0.05) was present for femur ash (day 16), with high phytase only increasing ash in challenged birds. The challenge decreased (*P* < 0.05) the BS of femur and tibia at each time point. Birds fed high dietary Ca had lower tibial Mg (*P* < 0.001), Fe (*P* < 0.001), Na (*P* < 0.001), and Zn (*P* < 0.05) concentrations (day 29). Altogether, high dietary Ca and phytase improved bone mineralization showing that attention to Ca and P nutrition and phytase matrix values is warranted when high levels of phytase are used.

## Introduction

Bone development is an important welfare and production concern in the commercial broiler industry. It is affected by a number of factors such as rapid growth rate (Julian, 1998), genetics (González-Cerón et al., 2015), management (Hester, 1994), nutrition (Saunders-Blades et al., 2009), and locomotion (Reiter and Bessei, 2009). Genetic selections have been carried out over the years to improve the growth performance in meat chickens without concurrent selection for a skeletal system that keeps up with increased body mass (Dibner, 2007). Genetic advances have led to a bird that achieves a given weight on less feed. However, the nutrient composition of diets, especially minerals for bone development, needs to be reassessed.

In the skeleton, hydroxyapatite (Ca_10_(PO4)_6_(OH)_2_) stores 99% of Ca and 80% of P of the body and is essential in bone development and mineralization. The remaining Ca is found in extracellular fluids, plasma, and within cells. Its functions include blood clotting, enzyme activation, neuromuscular function, muscle contraction, and intracellular signaling (Pilvi et al., 2008, Kozyreva et al., 2009, Vasin et al., 2010, Akbari Moghaddam Kakhki et al., 2018, Toyoda et al., 2018). Ca deficiency is not common in commercial chicken production, but there can be problems of malabsorption. For instance, high dietary phytate and fiber in the diet can interfere with Ca absorption (Selle et al., 2009, Wilkinson et al., 2011, dos Santos et al., 2014, Sadeghi et al., 2015). Dietary Ca may also cause malabsorption of trace minerals through precipitation. Mineral antagonisms in the digesta can cause a secondary deficiency in Zn, Cu, or Mn as Ca might outcompete these trace elements for transporters if fed at too high levels. (Shafey, 1993, Dibner et al., 2007).

The Ca requirement in modern broilers has been recently reported (Applegate et al., 2003, Li et al., 2012, Li et al., 2015, Li et al., 2016, Li et al., 2016, Abdulla et al., 2017, Kim et al., 2018b). The heightened interest in dietary Ca-related studies is due to its effect on phytase efficacy. Most broiler diets contain exogenous phytase, and their acceptance globally has led to the dietary reduction or exclusion of calcium phosphate and meat and bone meal. High dietary Ca in the form of limestone tends to elevate gut pH because of its acid-binding capacity and may cause Ca-phytate formation (Applegate et al., 2003). This chelate is refractory to phytase hydrolysis (Angel et al., 2002), thereby reducing the availability of Ca and phytate-bound P (Pallauf, 1994, Sebastian et al., 1996). It has been reported that exogenous phytases are beneficial only at relatively low dietary Ca levels and close Ca:P ratios (Beeson et al., 2017). For instance, there was a decrease in P retention in chickens fed a phytase-supplemented diet when the ratio of Ca:P was 2:1, but a higher P retention was recorded when the Ca:P ratio was narrowed to 1:1 (Aksakal and Bilal, 2002).

Calcium has been implicated in the outcome of necrotic enteritis (**NE**) because of changes in gut pH. Phytase may also play a role in NE as it affects Ca and P availability and breaking strength (**BS**) (Williams, 2005; Paiva et al., 2013; Yan et al., 2017). However, the effect of NE on bone mineralization is rarely reported in literature. Therefore, this study was designed to evaluate the effect of 2 levels of phytase and Ca (low or high) on serum Ca and P and bone mineralization in birds challenged and unchallenged by *Eimeria spp.* and *Clostridium perfringens*.

## Materials and methods

### Birds and Management

All experimental procedures were reviewed and approved by the University of New England's Animal Ethics Committee. A total of 768 chicks were weighed and randomly allocated to 48 floor pens (0.85 m^2^) with 6 replicate pens per treatment and 16 birds per pen. Softwood shavings were used as bedding material of about 8-cm deep in each pen. Each pen was fitted with a single tube feeder (32 cm in diameter) and 4 nipple drinkers. The lighting and temperature program during the experimental period followed the Ross 308 guidelines (Aviagen, 2014).

### Diet Composition

Diets were based on wheat, soybean meal, cold-pressed expeller canola meal and formulated to contain 3,000 kcal/kg MEn and 1.24% standard ileal digestible lysine in starter and 3,100 kcal/kg MEn and 1.14% and 0.99% standardized ileal digestible lysine in grower and finisher, respectively. Further details of diet composition are give in the study by Zanu et al., 2020. Four diets were formulated for each phase (starter, grower, finisher) in accordance with Ross 308 nutrient specifications (Aviagen, 2014). A 2 × 2 × 2 factorial arrangement of treatments was used. Factors were NE challenge (no or yes), Ca (0.6 or 1.0% for starter, 0.5 or 0.9% for grower, and 0.4 or 0.8% for finisher), and phytase (500 or 1500 FTU/kg, Quantum Blue; AB Vista, Marlborough, UK; both using 500 matrix values for Ca, P, Na, arginine, lysine, methionine, methionine + cystine, tryptophan, isoleucine, threonine, and valine of 1.65, 1.50, 0.35, 0.130, 0.170, 0.039, 0.390, 0.190, 0.255, 0.330, and 0.230 g/kg, respectively). A basal diet was produced for each Ca level and then divided in half, with appropriate phytase added to each over the top. Diets were then remixed and pelleted. The available P was held at the same level irrespective of the Ca level, that is, Ca/*P* = 0.6/0.4 vs 1.0/0.4 S; 0.5/0.36 vs 0.91/0.36 G; and 0.43/0.30 vs 0.83/0.30 F. Diet with high dietary Ca had a wider Ca/P (2.5), and those with lower dietary Ca had a narrower ratio (1.5). The diets were offered *ad libitum* throughout the starter (day 0–14), grower (day 14–28), and finisher (day 28–42) phases. The starter diets were offered in a crumbled form while the grower and finisher diets were mixed and pelleted at 65°C.

### Challenge

The NE challenge was performed in accordance with reported procedures (Stanley et al., 2014, Rodgers et al., 2015). Half of the birds (384) were challenged with 5,000 oocysts of field strains of *Eimeria acervulina* and *Eimeria maxima* and 2,500 oocytes of *Eimeria brunetti* (Eimeria Pty. Ltd., Ringwood North, VIC, Australia) on day 9 and 10^8^ CFU per mL of *C. perfringens* strain EHE-NE18 (known to express NetB toxin, Commonwealth Scientific and Industrial Research Organization, Geelong, Australia) on day 14 and day 15.

### Serum Ca, P, and Parathyroid Hormone

On day 16, blood samples were collected from 2 birds per pen after stunning and decapitation. The blood samples were centrifuged at 3,000 × *g* for 15 min, and the serum samples were collected. Serum calcium and phosphorus were determined by using Siemens Dimension Xpand Plus Autoanalyser (Siemens, Newark, NJ). The parathyroid hormone (PTH) was measured using the chicken PTH ELISA Kit (Abbexa Ltd., Cambridge Science Park, Cambridge, UK).

### Bone Traits

#### Bone Dimensions and Breaking Strength

On day 16 and day 29 after hatch, 2 birds per pen of average body weight were euthanized. Toe (middle), tibia, and femur were excised from the right leg of each bird. For the determination of BS, the tibia and femur were cleaned using a scalpel. The length (mm) (from the tip of the proximal end to the tip of the distal end) and width (mm) (at the medial region of the bone) of femur and tibia were measured using a Vernier caliper. They were then subjected to testing on a universal texture analyzer (Ametek Lloyd Instrument, Sussex, UK) set up with a 50-N load cell and a 3-point fixture bed at a test speed of 10 points of data per second. The Instron Blue Hill 3 software (Norwood, MA) was used to record the data. The force was applied to the midpoint of each tibia and femur with a 2-cm distance between the 2 fixed points supporting the bone.

#### Bone Ash Content

The toe and remnants of the femur and tibia were dried at 100°C in a forced-air oven (Watson Victor Ltd., Sydney, Australia) to a constant weight (around 24 h), weighed, and ashed at 600°C overnight in a muffle furnace (Carbolite, Sheffield, England) for determination of ash (%).

#### Bone Mineral Content

For the determination of minerals—Ca (%), P (%), K (%), Cu (%), Mg (%), Fe (mg/kg), Mn (mg/kg), Na (mg/kg), and Zn (mg/kg)—in the tibia, femur, and toe, approximately 1 g of ash were homogenized and digested in a Milestone Ultrawave Microwave (Milestone Srl, Sorisole, Italy) with nitric acid (HNO_3_). Minerals were measured on inductively coupled plasma emission spectrometer (Agilent, Victoria, Australia).

### Statistical Analyses

The data were evaluated as a fixed-effect model using the effects described in the statistical model, as follows:

$${\text{Y}}_{\text{ijkl}}=\mu +{\text{NE}}_{\text{i}}+{\text{P}}_{\text{j}}+{\text{Ca}}_{\text{k}}+{\left(\text{NE}*\text{P}\right)}_{\text{ij}}+{\left(\text{NE}*\text{Ca}\right)}_{\text{ik}}+{\left(\text{P}*\text{Ca}\right)}_{\text{jk}}+{\left(\text{NE}*\text{P}*\text{Ca}\right)}_{\text{ijk}}+{\text{e}}_{\text{ijkl}}$$

where *Y*_*ij*_ is the response expected independent variables, μ = overall mean, NE_i_ = fixed effect of NE (i = challenged or not challenged), P_j_ = the fixed effect of phytase (j = low or high), Ca_k_ = the fixed effect of calcium (k = low or high), (NE∗P)_ij_ = interaction between NE and phytase, (NE∗Ca)_ik_ = interaction between NE and Ca, (P∗Ca)_jk_ = interaction between phytase and calcium, (NE∗P∗Ca)_ijk_ = is the 3-way interaction, and *e*_*ij*_ is the random residual error ∼ *N* (0, s2e). The study used a completely randomized design with data analyzed as a 2 × 2 × 2 factorial arrangement of treatments using the Minitab 19 statistical software (State College, PA) to assess the main effects and 2- or 3-way interactions, with the factors as challenge (no or yes), Ca (low or high), and phytase (500 or 1500 FTU/kg). Tukey's mean separation test was used to make pairwise comparisons between treatment means (*P* < 0.05). The Box-Cox transformation of the Minitab 19 statistical software was used to test and confirm the normality of all the data before analysis. The statistical unit was the average of the 2 birds.

## Results

### Serum Ca^+^ and P on day 16

A challenge × phytase interaction was observed for serum Ca^+^ and P, where in the nonchallenged group, increasing phytase from 500 to 1500 FTU resulted in lower serum Ca^+^ (*P* < 0.05), and the serum P was also lower when challenged than when nonunchallenged regardless of the level of phytase (Table 1). The challenge also decreased serum Ca^+^ regardless of the level of phytase. A challenge × dietary Ca interaction was detected for serum Ca^+^, in that, there was more serum Ca in the unchallenged birds fed high dietary Ca and less serum Ca in the challenged birds fed low dietary Ca than any other treatment. Challenged birds at both Ca levels had comparable serum Ca. The challenge as a main effect decreased the serum P irrespective of the level of phytase and Ca. High dietary Ca as a main effect decreased serum P (*P* < 0.001). The PTH values were not affected (*P* > 0.05) by any of the factors.

**Table 1:** Effect of necrotic enteritis phy and Ca on serum Ca and P, day 16.

Effects				Serum Ca (mg/dL)	Serum phosphorus (mg/dL)	PTH (ng/mL)
	NE	Phy	Ca			
2-Way interactions						
NE∗Phy	−	500		9.69^a^	8.17^a^	1.47
	−	1,500		9.08^b^	8.93^a^	1.01
	+	500		8.35^c^	6.40^b^	2.26
	+	1,500		8.40^c^	6.15^b^	1.37
NE∗Ca						
	−		Low	8.98^b^	9.04	0.96
	−		High	9.79^a^	8.05	1.53
	+		Low	8.26^c^	6.73	1.61
	+		High	8.48^b,c^	5.83	2.01
Main effects						
NE	−			9.38	8.55^a^	1.24
	+			8.37	6.28^b^	1.81
Ca			Low	8.62	7.89^a^	1.28
			High	9.13	6.94^b^	1.77
SEM				0.52	0.77	0.96
*P* value						
NE				0.001	0.001	0.328
Phy				0.055	0.277	0.251
Ca				0.001	0.001	0.405
NE × Phy				0.026	0.035	0.710
NE × Ca				0.043	0.829	0.884
Phy × Ca				0.319	0.710	0.179
NE × Phy × Ca				0.620	0.131	0.371

^a-c^Different superscript alphabets in the same column means there is significant difference (*P* < 0.05).

Abbreviations: Ca, calcium; NE, necrotic enteritis; phy, phytase; PTH, parathyroid hormone.

Phytase = Quantum Blue 5G.

2- or 3-Way interaction by Tukey.

### Bone Traits, day 16

At day 16, a challenge × Ca interaction was present for tibial BS (Table 2), showing no difference between unchallenged birds fed low Ca and challenged birds fed high Ca, but unchallenged birds fed high Ca had significantly greater BS, and challenged birds fed low Ca had lower BS than all other treatments. A challenge × phytase interaction was detected for femur ash on day 16 (Table 3) (*P* < 0.05), indicating that challenged birds fed low phytase had lower femur ash than those fed other treatment diets. Tibial BS increased (*P* < 0.001) in the birds fed high phytase diet. Tibial length (*P* < 0.01) and width (*P* < 0.05) and femur length (*P* < 0.05) were decreased by the challenge (Table 2). High Ca as a main effect increased femur width (*P* < 0.05; Table 2) and toe ash (*P* < 0.001; Table 3). Ash content of toe (*P* < 0.01), femur (*P* < 0.05), and tibia (*P* < 0.001) were increased by high dietary Ca. High phytase as a main effect increased tibial ash (*P* < 0.001).

**Table 2:** Effect of necrotic enteritis, phy, and Ca on bone traits, day 16.

Effects				Tibial length (mm)	Femur length (mm)	Tibial width (mm)	Femur width (mm)	Tibial breaking strength (N)
	NE	Phy	Ca					
2-Way interactions								
NE∗Ca								
	−		Low	56.61	43.27	4.38	5.34	94.23^b^
	−		High	56.27	43.05	4.50	5.70	131.17^a^
	+		Low	55.26	42.54	4.26	5.33	71.28^c^
	+		High	54.74	41.98	4.26	5.43	91.80^b^
Main effects								
NE	−			56.44^a^	43.16^a^	4.44^a^	5.52	112.70
	+			55.00^b^	42.26^b^	4.26^b^	5.37	81.54
Phy		500		55.41	42.48	4.29	5.39	91.60^b^
		1,500		56.03	42.95	4.42	5.51	102.64^a^
Ca			Low	55.94	42.90	4.32	5.34^b^	82.75
			High	55.50	42.52	4.38	5.57^a^	111.48
SEM				1.12	0.71	0.10	0.18	10.16
*P* value								
NE				0.002	0.012	0.020	0.171	0.001
Phy				0.160	0.178	0.091	0.230	0.001
Ca				0.326	0.266	0.456	0.033	0.001
NE × Phy				0.784	0.967	0.281	0.606	0.932
NE × Ca				0.832	0.611	0.427	0.211	0.011
Phy × Ca				0.946	0.755	0.068	0.087	0.527
NE × Phy × Ca				0.429	0.717	0.569	0.969	0.222

^a-c^Different superscript alphabets in the same column means there is significant difference (*P* < 0.05).

Abbreviations: Ca, calcium; NE, necrotic enteritis; phy, phytase.

Phytase = Quantum Blue 5G.

2- or 3-Way interaction by Tukey.

**Table 3:** Effect of necrotic enteritis, phy, and Ca on bone ash (%), day 16 and 29.

Effects				day 16			day 29		
				Toe ash	Femur ash	Tibial ash	Toe ash	Femur ash	Tibial ash
	NE	Phy	Ca						
2-Way interactions									
NE∗Phy	−	500		11.17	48.69^a^	47.77	11.60	46.32	48.07
	−	1,500		11.34	49.26^a^	48.85	11.77	47.02	49.01
	+	500		11.00	46.62^b^	46.96	11.66	46.69	48.74
	+	1,500		11.04	49.01^a^	48.69	11.87	48.65	48.74
Main effects									
Phy		500		11.09	47.66	47.37^b^	11.63	46.50	48.41
		1,500		11.22	49.14	48.77^a^	11.82	47.84	48.87
Ca			Low	10.75^a^	46.42^b^	46.20^b^	11.37^b^	46.21^b^	47.51^b^
			High	11.55^b^	50.37^a^	49.94^a^	12.08^a^	48.12^a^	49.76^a^
SEM				0.33	1.00	0.89	0.44	2.96	0.75
*P* value									
NE				0.150	0.003	0.210	0.711	0.189	0.569
Phy				0.467	0.001	0.001	0.390	0.082	0.181
Ca				0.001	0.001	0.001	0.002	0.015	0.001
NE × Phy				0.631	0.016	0.397	0.916	0.402	0.179
NE × Ca				0.329	0.138	0.899	0.338	0.114	0.068
Phy × Ca				0.603	0.197	0.141	0.737	0.454	0.688
NE × Phy × Ca				0.656	0.580	0.840	0.558	0.219	0.458

^a,b^Different superscript alphabets in the same column means there is significant difference (*P* < 0.05).

Abbreviations: Ca, calcium; NE, necrotic enteritis; phy, phytase.

Phytase = Quantum Blue 5G.

2- or 3-Way interaction by Tukey.

### Bone Traits, day 29

At day 29, a challenge × phytase interaction was detected for tibial length, while in the nonchallenged birds, high phytase increased (*P* < 0.05) tibial length compare to those on low phytase (Table 4). The challenged birds recorded lower tibial length regardless of the level of phytase. The challenge decreased tibial width (*P* < 0.001), tibial BS (*P* < 0.001), femur width (*P* < 0.001), and femur BS (*P* < 0.001). High phytase as a main effect increased femur length (*P* < 0.01) and femur BS (*P* < 0.01). High Ca as a main effect increased tibial BS (*P* < 0.001), femur BS (*P* < 0.001), tibial ash (*P* < 0.001), femur ash (*P* < 0.01), and toe ash (*P* < 0.01) (Table 3).

**Table 4:** Effect of necrotic enteritis, phy, and Ca on bone traits, day 29.

Effects				Tibial length (mm)	Femur length (mm)	Tibial width (mm)	Femur width (mm)	Tibial breaking strength (N)	Femur breaking strength (N)
	NE	Phy	Ca						
2-Way interactions									
NE∗Phy	−	500		77.65^b^	58.51	6.62	8.20	366	243
	−	1,500		79.69^a^	60.03	6.78	8.31	389	264
	+	500		75.56^c^	57.37	6.25	7.67	310	202
	+	1,500		75.79^c^	57.94	6.23	7.76	325	233
Main effects									
NE	−			78.67	59.27^a^	6.70^a^	8.25^a^	377^a^	254^a^
	+			75.68	57.66^b^	6.24^b^	7.71^b^	317^b^	218^b^
Phy		500		76.60	57.94^b^	6.43	7.93	338	222^b^
		1,500		77.74	58.98^a^	6.51	8.03	357	249^a^
Ca			Low	77.58^a^	58.70	6.48	7.89	319^b^	199^b^
			High	76.76^b^	58.22	6.46	8.07	376^a^	272^a^
SEM				1.08	1.01	0.22	0.21	28.16	22.45
*P* value									
NE				0.001	0.001	0.001	0.001	0.001	0.001
Phy				0.005	0.009	0.429	0.280	0.178	0.009
Ca				0.036	0.212	0.799	0.051	0.001	0.001
NE × Phy				0.021	0.220	0.322	0.889	0.739	0.594
NE × Ca				0.174	0.349	0.505	0.441	0.790	0.706
Phy × Ca				0.406	0.393	0.102	0.379	0.844	0.719
NE × Phy × Ca				0.487	0.833	0.973	0.691	0.503	0.572

^a,b^Different superscript alphabets in the same column means there is significant difference (*P* < 0.05).

Abbreviations: Ca, calcium; NE, necrotic enteritis; phy, phytase.

Phytase = Quantum Blue 5G.

2- or 3-Way interaction by Tukey.

### Toe Mineral Concentration, day 16

At day 16, a challenge × phytase interaction was present for Mg, while in the challenge group, high phytase decreased (*P* < 0.05) Mg in the toe compare to birds on low phytase (Table 5). A challenge × Ca interaction was also observed for Na indicating that in the nonchallenge group, low dietary Ca increased (*P* < 0.05) the Na content compare to those fed high dietary Ca. A strong tendency of phytase × Ca interaction was observed for Zn (*P* = 0.051), that is, in birds fed high phytase, low dietary Ca increased Zn concentration compare to those on high Ca diet. Challenge as main effect decreased K (*P* < 0.001) and Zn (*P* < 0.001). High dietary Ca as the main effect decreased the K (*P* < 0.01), Mg (*P* < 0.001), and Mn (*P* < 0.001) deposition.

**Table 5:** Effect of necrotic enteritis, phy, and Ca on toe mineral concentration, day 16.

Effects				Ca, %	P, %	K, %	Mg, %	Mn, mg/kg	Na, mg/kg	Zn, mg/kg
	NE	Phy	Ca							
2-Way interactions										
NE∗Phy	−	500		32.10	16.91	3.00	1.01^a^	43.43	4.56	507
	−	1,500		32.52	17.19	2.89	1.01^a^	43.94	4.49	518
	+	500		32.45	16.95	2.42	0.99^a^	50.91	4.31	462
	+	1,500		32.09	16.58	2.25	0.92^b^	47.28	4.18	462
NE∗Ca										
	−		Low	31.90	17.18	3.06	1.09	52.30	4.76^a^	521
	−		High	32.72	16.91	2.83	0.94	35.07	4.29^b^	504
	+		Low	32.15	16.90	2.42	1.03	57.10	4.26^b^	465
	+		High	32.39	16.64	2.26	0.89	41.10	4.23^b^	459
Phy∗Ca										
		500	Low	31.82	16.97	2.78	1.07	57.09	4.50	481^b^
		500	High	32.73	16.89	2.64	0.93	37.25	4.37	488^b^
		1,500	Low	32.24	17.11	2.71	1.05	52.31	4.52	505^a^
		1,500	High	32.37	16.67	2.44	0.09	38.91	4.15	475^c^
Main effects										
NE	−			32.31	17.05	2.95^a^	1.01	43.68	4.52	512^a^
	+			32.26	16.76	2.34^b^	0.96	49.10	4.24	462^b^
Ca			Low	32.03	17.04	2.74^a^	1.06^a^	54.70^a^	4.51	493
			High	32.55	16.78	2.54^b^	0.92^b^	38.08^b^	4.26	482
SEM				1.23	0.51	0.22	0.03	9.50	0.22	21.63
*P* value										
NE				0.928	0.155	0.001	0.001	0.074	0.005	0.001
Phy				0.947	0.824	0.053	0.044	0.600	0.293	0.553
Ca				0.253	0.179	0.006	0.001	0.001	0.010	0.213
NE × Phy				0.385	0.099	0.625	0.020	0.486	0.711	0.548
NE × Ca				0.526	0.982	0.591	0.594	0.836	0.022	0.539
Phy × Ca				0.397	0.361	0.336	0.845	0.282	0.189	0.051
NE × Phy × Ca				0.360	0.273	0.447	0.902	0.392	0.490	0.432

^a,b^Different superscript alphabets in the same column means there is significant difference (*P* < 0.05).

Abbreviations: Ca, calcium; NE, necrotic enteritis; phy, phytase.

Phytase = Quantum Blue 5G.

2- or 3-Way interaction by Tukey.

### Toe Mineral Concentration, day 29

A challenge × dietary Ca interaction was detected for toe Ca, that is, in the challenge birds, high dietary Ca increased (*P* < 0.05) toe Ca content (Table 6) to a level greater than all other treatments. A challenge × Ca interaction was detected for Mg, Mn, and Na, showing that in the challenge birds, high dietary Ca decreased the contents of Mg (*P* < 0.05) and Na (*P* < 0.05). High dietary Ca decreased Mn in both challenged and unchallenged. A phytase × dietary Ca interaction was also detected for Mn and Zn where high dietary Ca decreased (*P* < 0.05) Mn in both low and high phytase groups. In birds fed high phytase, the Zn (*P* < 0.05) deposition was decreased by high dietary Ca compared to those fed low Ca diet. Challenge as a main effect increased P (*P* < 0.001) and Zn (*P* < 0.001) concentrations. High Ca as a main effect decreased (*P* < 0.01) K concentration.

**Table 6:** Effect of necrotic enteritis, phy, and Ca on toe mineral concentration, day 29.

Effects				Ca, %	P, %	K, %	Mg, %	Mn, mg/kg	Na, mg/kg	Zn, mg/kg
	NE	Phy	Ca							
2-Way interactions										
NE∗Ca										
	−		Low	32.74^b^	16.58	2.27	0.91^b^	46.93^a^	4.71^a^	486
	−		High	33.20^b^	16.57	2.16	0.87^b^	38.65^b^	4.61^a,b^	451
	+		Low	33.36^b^	17.00	2.35	0.98^a^	53.41^a^	4.86^a^	496
	+		High	34.81^a^	17.07	2.14	0.88^b^	34.87^b^	4.32^b^	518
Phy∗Ca										
		500	Low	32.72	16.62	2.27	0.93	44.88^b^	4.74	480^b^
		500	High	34.10	16.84	2.19	0.87	37.16^c^	4.50	476^b^
		1,500	Low	33.38	16.96	2.35	0.96	55.46^a^	4.82	525^a^
		1,500	High	33.91	16.80	2.11	0.88	36.36^c^	4.43	472^b^
Main effects										
NE	−			32.97	16.58^b^	2.21	0.89	42.79	4.65	469^b^
	+			34.09	17.04^a^	2.25	0.93	44.14	4.59	507^a^
Ca			Low	33.05	16.79	2.31^a^	0.95	50.17	4.78	502
			High	34.01	16.82	2.15^b^	0.87	36.76	4.46	474
SEM				0.54	0.26	0.09	0.03	4.64	0.20	29.76
*P* value										
NE				0.001	0.001	0.466	0.008	0.519	0.450	0.001
Phy				0.324	0.171	0.915	0.215	0.023	0.984	0.050
Ca				0.001	0.799	0.002	0.001	0.001	0.001	0.008
NE × Phy				0.872	0.644	0.232	0.568	0.124	0.244	0.146
NE × Ca				0.042	0.740	0.294	0.020	0.018	0.014	0.512
Phy × Ca				0.075	0.070	0.122	0.473	0.009	0.357	0.019
NE × Phy × Ca				0.754	0.724	0.489	0.720	0.697	0.628	0.082

^a-c^Different superscript alphabets in the same column means there is significant difference (*P* < 0.05).

Abbreviations: Ca, calcium; NE, necrotic enteritis; phy, phytase.

Phytase = Quantum Blue 5G.

2- or 3-Way interaction by Tukey.

### Femur Mineral Concentration, day 16

At day 16, the challenge as main effect decreased Ca (*P* < 0.01) and Cu (*P* < 0.001) content while it increased K (*P* < 0.001) and Mn (*P* < 0.001) content (Table 7) with no interaction *P* > 0.05). High phytase as a main effect decreased K concentration (*P* < 0.01) but increased Mn (*P* < 0.05) content with no interaction. High dietary Ca as a main effect increased (*P* < 0.001) femur Ca content while it decreased that of P (*P* < 0.01), K (*P* < 0.001), Mg (*P* < 0.001), Fe (*P* < 0.05), Mn (*P* < 0.001), and Na (*P* < 0.01).

**Table 7:** Effect of necrotic enteritis, phy, and Ca on femur mineral concentration, day 16.

Effects				Ca, %	P, %	K, %	Mg, %	Fe, mg/kg	Mn, mg/kg	Na, mg/kg
	NE	Phy	Ca							
Main effects										
NE	−			38.31^a^	18.07	0.79^b^	1.00	366	20.83^b^	1.06^b^
	+			37.86^b^	18.04	1.02^a^	1.02	359	32.92^a^	1.34^a^
Phy		500		38.09	18.08	0.95^a^	1.02	363	25.60^b^	1.22
		1,500		38.09	18.03	0.86^b^	1.00	361	28.14^a^	1.19
Ca			Low	37.77^b^	18.18^a^	1.01^a^	1.08^a^	392^a^	31.19^a^	1.26^a^
			High	38.41^a^	17.93^b^	0.80^b^	0.94^b^	331^b^	22.56^b^	1.14^b^
SEM				0.38	0.16	0.12	0.04	88.20	4.34	0.12
*P* value										
NE				0.003	0.755	0.001	0.327	0.805	0.001	0.001
Phy				0.997	0.521	0.003	0.254	0.892	0.023	0.464
Ca				0.001	0.004	0.001	0.000	0.016	0.001	0.002
NE × Phy				0.116	0.975	0.151	0.089	0.499	0.271	0.812
NE × Ca				0.275	0.097	0.581	0.709	0.176	0.139	0.472
Phy × Ca				0.286	0.364	0.888	0.911	0.196	0.137	0.339
NE × Phy × Ca				0.315	0.925	0.257	0.261	0.100	0.445	0.873

^a,b^Different superscript alphabets in the same column means there is significant difference (*P* < 0.05).

Abbreviations: Ca, calcium; NE, necrotic enteritis; phy, phytase.

Phytase = Quantum Blue 5G.

2- or 3-Way interaction by Tukey.

### Femur Mineral Concentration, day 29

A challenge × dietary Ca interaction was detected for femur Ca concentration, indicating that high dietary Ca increased (*P* < 0.05) femur Ca only in the nonchallenge group (Table 8). A challenge × dietary Ca interaction was detected for P, Mg, and Na concetrations in femur, where high dietary Ca decreased the concentration of P (*P* < 0.05) in the challenged. The Mg content was decreased (*P* < 0.05) in both challenged and unchallenged groups fed high dietary Ca. The Na content decreased (*P* < 0.05) only in the challenge group fed high dietary Ca. A phytase × Ca interaction was detected for Mn concentration in femur, where the Mn decreased (*P* < 0.001) in both low- and high-phytase groups fed high dietary Ca. A phytase × Ca interaction was detected for Zn concentration in femur, where high Ca decreased (*P* < 0.001) Zn level only in the presence of high phytase. Challenge as a main effect increased Fe (*P* < 0.001) and Na (*P* < 0.001) contents but decreased that of Mn (*P* < 0.05). Birds on high dietary Ca had lower K (*P* < 0.001) and Fe (*P* < 0.001) concentrations.

**Table 8:** Effect of necrotic enteritis, phy, and Ca on femur mineral concentration, day 29.

Effects				Ca, %	P, %	K, %	Mg, %	Fe, mg/kg	Mn, mg/kg	Na, mg/kg	Zn, mg/kg
	NE	Phy	Ca								
2-Way interactions											
NE∗Ca											
	−		Low	39.14^b^	18.50^a,b^	0.85	1.00^a^	500	29.42	1.13^a^	496
	−		High	40.01^a^	18.44^b^	0.64	0.92^b^	378	18.66	1.08^a^	483
	+		Low	39.02^b^	18.50^a^	0.98	1.01^a^	566	27.92	1.07^a^	502
	+		High	39.32^b^	18.13^b^	0.79	0.89^b^	419	16.25	0.92^b^	476
Phy∗Ca											
		500	Low	38.97	18.43	0.91	1.01	536	25.50^b^	1.11	462^b^
		500	High	39.77	18.29	0.73	0.90	406	16.95^c^	1.10	475^b^
		1,500	Low	39.19	18.57	0.92	1.02	530	31.84^a^	1.07	536^a^
		1,500	High	39.55	18.28	0.70	0.91	392	17.96^c^	1.00	484^b^
Main effects											
NE	−			39.57	18.47	0.75^b^	0.96	439^b^	24.04^a^	0.99	490
	+			39.17	18.31	0.89^a^	0.96	492^a^	22.09^b^	1.10	489
Ca			Low	39.08	18.50	0.92^a^	1.02	533^a^	28.67	1.10	499
			High	39.66	18.29	0.72^b^	0.91	399^b^	17.46	1.00	480
SEM				0.30	0.12	0.05	0.02	21.63	1.60	0.05	16.08
*P* value											
NE				0.007	0.032	0.001	0.989	0.001	0.012	0.001	0.960
Phy				0.970	0.353	0.580	0.330	0.332	0.000	0.543	0.000
Ca				0.001	0.005	0.001	0.001	0.001	0.001	0.001	0.022
NE × Phy				0.122	0.146	0.925	0.381	0.721	0.682	0.950	0.316
NE × Ca				0.048	0.035	0.751	0.027	0.239	0.546	0.020	0.397
Phy × Ca				0.124	0.278	0.364	0.840	0.701	0.001	0.601	0.001
NE × Phy × Ca				0.295	0.489	0.183	0.959	0.808	0.511	0.717	0.815

^a-c^Different superscript alphabets in the same column means there is significant difference (*P* < 0.05).

Abbreviations: Ca, calcium; NE, necrotic enteritis; phy, phytase.

Phytase = Quantum Blue 5G.

2- or 3-Way interaction by Tukey.

### Tibial Mineral Concentration, day 16

At day 16, tibia concentration of Mn (*P* < 0.001) and Na (*P* < 0.001) was greater in challenged than in unchallenged birds (Table 9). High phytase increased Mn (*P* < 0.05) and Zn (*P* < 0.01) concentration in tibia but decreased K (*P* < 0.05). High dietary Ca increased (*P* < 0.05) the Ca concentration in tibia but decreased K (*P* < 0.001), Fe (*P* < 0.001), Mn (*P* < 0.001), and Na (*P* < 0.01) concentration.

**Table 9:** Effect of necrotic enteritis, phy, and Ca on tibial mineral concentration, day 16.

Effects				Ca, %	K, %	Fe, mg/kg	Mn, mg/kg	Na, mg/kg	Zn, mg/kg
	NE	Phy	Ca						
Main effects									
NE	−			38.07	0.95	352	20.92^b^	1.45^b^	466
	+			38.55	0.95	358	31.79^a^	1.60^a^	462
Phy		500		38.02	0.98^a^	362	25.20^b^	1.54	450^b^
		1,500		38.62	0.92^b^	348	27.51^a^	1.51	478^a^
Ca			Low	37.78^b^	1.05^a^	395^a^	30.65^a^	1.57^a^	463
			High	38.84^a^	0.85^b^	315^b^	22.06^b^	1.48^b^	465
SEM				0.97	0.06	20.24	3.44	0.08	21.27
*P* value									
NE				0.138	0.836	0.592	0.001	0.001	0.682
Phy				0.054	0.049	0.123	0.024	0.386	0.006
Ca				0.002	0.001	0.001	0.001	0.007	0.813
NE × Phy				0.879	0.058	0.865	0.091	0.182	0.093
NE × Ca				0.459	0.327	0.286	0.364	0.119	0.693
Phy × Ca				0.857	0.648	0.357	0.124	0.488	0.192
NE × Phy × Ca				0.573	0.419	0.587	0.777	0.594	0.658

^a,b^Different superscript alphabets in the same column means there is significant difference (*P* < 0.05).

Abbreviations: Ca, calcium; NE, necrotic enteritis; phy, phytase.

Phytase = Quantum Blue 5G.

2- or 3-Way interaction by Tukey.

### Tibial Mineral Concentration, day 29

A phytase × Ca interaction was detected for Mn, where high dietary Ca decreased (*P* < 0.01) Mn concentration in tibia of both low- and high-phytase groups (Table 10). Challenged birds recorded higher K (*P* < 0.01) and Fe (*P* < 0.05) concentrations in tibia. Birds fed diets with high phytase had higher (*P* < 0.001) Zn concentration in tibia. Birds fed diets with high dietary Ca had lower K (*P* < 0.001), Mg (*P* < 0.001), Fe (*P* < 0.001), Na (*P* < 0.001), and Zn (*P* < 0.05) concentrations in tibia.

**Table 10:** Effect of necrotic enteritis, phy, and Ca on tibial mineral concentration, day 29.

Effects				Ca, %	P, %	K, %	Mg, %	Fe, mg/kg	Mn, mg/kg	Na, mg/kg	Zn, mg/kg
	NE	Phy	Ca								
2-Way interactions											
Phy∗Ca											
		500	Low	39.31	18.37	0.90	1.00	455	23.35^b^	1.04	446
		500	High	38.70	17.70	0.71	0.86	341	15.27^c^	0.90	434
		1,500	Low	39.44	18.48	0.91	1.00	444	29.56^a^	1.02	501
		1,500	High	39.56	18.17	0.73	0.90	345	16.88^c^	0.93	462
Main effects											
NE	−			39.50	18.26	0.78^b^	0.93	382^b^	21.53	0.98	457
	+			39.01	18.1	0.83^a^	0.94	410^a^	20.99	0.97	464
Phy		500		39.00	18.04	0.81	0.93	398	19.31	0.97	440^b^
		1,500		39.50	18.32	0.82	0.95	394	23.22	0.98	481^a^
Ca			Low	39.37	18.42	0.91^a^	1.00^a^	449^a^	26.45	1.03^a^	474^a^
			High	39.13	17.94	0.72^b^	0.88^b^	343^b^	16.07	0.92^b^	448^b^
SEM				0.40	0.97	0.04	0.05	22.65	1.24	0.06	28.29
*P* value											
NE				0.391	0.528	0.004	0.517	0.022	0.464	0.909	0.525
Phy				0.377	0.271	0.528	0.126	0.759	0.001	0.843	0.001
Ca				0.668	0.061	0.001	0.001	0.001	0.001	0.001	0.020
NE × Phy				0.235	0.222	0.507	0.237	0.401	0.650	0.999	0.052
NE × Ca				0.451	0.353	0.535	0.091	0.235	0.537	0.104	0.481
Phy × Ca				0.520	0.484	0.802	0.265	0.529	0.003	0.215	0.208
NE × Phy × Ca				0.349	0.326	0.286	0.247	0.967	0.630	0.210	0.860

^a-c^Different superscript alphabets in the same column means there is significant difference (*P* < 0.05).

Abbreviations: Ca, calcium; NE, necrotic enteritis; phy, phytase.

Phytase = Quantum Blue 5G.

2- or 3-Way interaction by Tukey.

## Discussion

### Serum Ca and P

Meeting the Ca requirement of chickens is essential for many biochemical processes and bone formation. In this study, the challenge decreased both serum Ca and P regardless of the level of phytase or dietary Ca. A reduction in serum Ca and P in birds during disease has been previously reported (Fernandez et al., 1994, Yarru et al., 2009, Zhao et al., 2010, Yunus and Böhm, 2013, Igwe et al., 2018). The current observation might be due to the low feed intake and poor P digestibility as a result of poor gut integrity and increased endogenous losses that characterize NE incidence and was the case in the present study (Zanu et al., 2019). On the other hand, the high Ca diet depressed serum P and increased serum Ca only under the unchallenged condition, suggesting again that the serum was reflecting the imbalanced Ca:P absorption taking place from the intestine. Diets with wide Ca:P ratios have been shown to depress serum P (Sebastian et al., 1996, Bilal et al., 2015). The present findings agree with a recent observation in ducklings in which a higher serum Ca^+^ concentration was observed in the diet with 0.95% Ca level (Zhu et al., 2018). In that study, a low P diet led to a decline in serum P concentration as dietary Ca increased from 0.55 to 0.95%. This suggests that high dietary Ca might precipitate P and inhibit P absorption.

In the unchallenged birds, higher dietary phytase decreased serum Ca while it increased serum P, suggesting the phytase released more P than Ca. This finding agrees with the report that phytase supplementation decreases serum Ca^+^ in chickens (Viveros et al., 2002). There was no detectable effect of the treatments on PTH in this study.

### Bone Mineralization

Skeletal integrity is commonly used as an indicator for mineral adequacy in broiler diets. Dietary inorganic nutrients are relevant to broiler bone development and mineralization. The interaction between dietary Ca and bone traits has been previously reported. Increase in bone BS and ash of chicks fed diets containing 1.25% compared with 1.00% of Ca were found by Abdulla et al. (2017). Increased tibial ash as a result of increasing dietary Ca has been reported (Watkins and Southern, 1991, Onyango et al., 2003). These reports agree with the results of the present study, in that, with high dietary Ca, tibial BS, femur Ca, and toe, tibia, and femur ash were increased in both challenged and unchallenged birds. However, the findings of the present study revealed that during NE challenge, high dietary Ca may not improve the deposition of minerals other than Ca into the bone. The present study shows that minerals such as Mg, Mn, Na, and P were highly concentrated in the bone of birds fed low dietary Ca relative to those on high dietary Ca under challenge condition. For instance, the decrease in femur P on day 16 in birds fed high Ca diet might be because the high Ca depressed P availability from the diet, and this is reflected in both serum and bone P levels.

Li et al. (2012) also reported a significant decrease in tibial mineral composition in broilers fed a diet of wider Ca/P (Ca/nPP = 4.1) than in those on a narrower ratio (Ca/nPP = 2.2). Again, it is most probable that the wider Ca and P ratio led to the precipitation of these minerals, reducing their bioavailability (Saripinar-Aksu et al., 2012). That observation could be because excess Ca binds with phytate to form an insoluble complex that is less accessible to phytase. Also, a high concentration of dietary Ca relative to available P increases the chances of trace minerals competing for transporters, thereby leading to deficiency (Yan et al., 2005, Plumstead et al., 2008). The NE challenge and high Ca might have also resulted in a doubly depressive effect on the absorption of other minerals due to mucosal damage.

There are numerous reports on the benefits of phytase in improving bone characteristics and mineralization (Viveros et al., 2002, Gautier et al., 2018). In this study, high phytase improved femur ash in both unchallenged and challenged birds. High phytase increased bone length, width, BS, ash, and mineral concentration. Phytic acid decreases the availability of minerals as it has a strong affinity for multivalent metals such as Fe, Zn, and Ca. The use of phytases, especially at higher doses, hydrolyzes the phytate molecules and releases these ions which are important in bone formation. Therefore, phytase supplementation has been reported to improve bone traits and mineralization in many studies (Rutherfurd et al., 2012, Olukosi and Fru-Nji, 2014, Kim et al., 2018a, Leyva-Jimenez et al., 2018).

In the present study, the challenge decreased the bone lengths, widths, and BSs at each time point after hatch. In a similar challenge study, the ash and Ca content of tibia of broilers were decreased when chickens were infected with salmonella, but the inclusion of probiotics (*Bacillus subtilis*) to the diets of the challenged chicks compensated for the negative effects of the challenge (Sadeghi, 2014). Notwithstanding, there was an increase in the concentration of some minerals in the challenge birds. Intestinal epithelial tight junction disruption might have increased the paracellular absorption of these minerals. However, there is little information on the paracellular absorption of trace minerals as compared to Ca. Thus, Bronner (1998) was cautious in suggesting that all minerals were absorbed through the paracellular pathway. Increased mineral concentration in NE challenged birds, therefore, warrants further research.

In conclusion, the toe, femur, and tibia responded differently to the challenge, Ca and phytase levels. Nonetheless, generally, higher Ca benefited many measures of bone integrity regardless of challenge or phytase dose. The higher phytase dose was equivocal, and this may have been due to those diets having too much available P compared with Ca.
